# Uricase alkaline enzymosomes with enhanced stabilities and anti-hyperuricemia effects induced by favorable microenvironmental changes

**DOI:** 10.1038/srep20136

**Published:** 2016-01-29

**Authors:** Yunli Zhou, Mi Zhang, Dan He, Xueyuan Hu, Huarong Xiong, Jianyong Wu, Biyue Zhu, Jingqing Zhang

**Affiliations:** 1 Chongqing Research Center for Pharmaceutical Engineering, Chongqing Medical University, Yixueyuan Road, Yuzhong District, Chongqing 400016, China; 2 West China School of Pharmacy, Sichuan University, Chengdu 610041, China

## Abstract

Enzyme therapy is an effective strategy to treat diseases. Three strategies were pursued to provide the favorable microenvironments for uricase (UCU) to eventually improve its features: using the right type of buffer to constitute the liquid media where catalyze reactions take place; entrapping UCU inside the selectively permeable lipid vesicle membranes; and entrapping catalase together with UCU inside the membranes. The nanosized alkaline enzymosomes containing UCU/(UCU and catalase) (ESU/ESUC) in bicine buffer had better thermal, hypothermal, acid-base and proteolytic stabilities, *in vitro* and *in vivo* kinetic characteristics, and uric acid lowering effects. The favorable microenvironments were conducive to the establishment of the enzymosomes with superior properties. It was the first time that two therapeutic enzymes were simultaneously entrapped into one enzymosome having the right type of buffer to achieve added treatment efficacy. The development of ESU/ESUC in bicine buffer provides valuable tactics in hypouricemic therapy and enzymosomal application.

An enzyme therapy^1^,^2^,^3^,^4^ such as uricase (UCU) application represents an alternative strategy which is more effective than the traditional chemotherapy. The incidences of hyperuricaemia and its related diseases have been growing fast worldwide^5^,^6^. The commonly used allopurinol and febuxostat are ineffective dissolving existing uric acid crystals, and their long-term use may disturb the purine metabolism^7^. UCU is effective at dissolving existing uric acid by converting uric acid into more soluble allantoin. UCU has been used in the clinical detection of uric acid and treatment of hyperuricemia for over 40 years^8^,^9^. It has also been used for the prevention and treatment of tumor lysis syndrome^10^. Recently, a Phase I study of UCU in the reduction of acute graft-versus-host disease after myeloablative allogeneic stem cell transplantation has been conducted^11^.

However, more extensive use of UCU has been hampered by its disadvantages ascribed to the intrinsic properties of an enzyme, such as relatively low catalytic activity and therapeutic effect under physical conditions. Therefore, there is an urgent need to develop superior UCU delivery systems with enhanced stabilities and catalytic activities when administered *in vivo*. A few efforts have been made to make UCU better: developing new UCU or recombinant UCU with higher activity using the mutation method^12^,^13^; immobilizing in polymer polypyrrole-polyvinyl sulphonate film to obtain new biosensors^14^; conjugating with polymer polyethylene glycol to achieve sustained effects and improve enzymatic stabilities^15^; and encapsulating in alkaline enzymosomes (i.e., nanosized liposomes containing enzyemes) to enhance biochemical and pharmacological characteristics^16^,^17^. Particularly, a recent report from our laboratory showed that the enzymosome containing UCU (ESU) has the potential to unexpectedly improve the stability and therapeutic efficacy of UCU^18^.

Here, we design and develop the novel nanosized delivery systems, alkaline enzymosomes containing UCU and catalase (CAT) (ESUC), which have better stabilities and anti-hyperuricemia effects than above-mentioned ESU. Our design is based on the hypothesis that changes of the microenvironment where the enzymatic reactions take place may induce the conformational changes of enzymes, accompanied by varied catalytic activities. The therapeutic efficacy of an enzyme is closely related to its catalytic activity, which may vary as its conformation changes in different microenvironments^19^. Three strategies are pursued to provide the favorable microenvironment for UCU to eventually improve its catalytic performance: using the right type of buffer to constitute the liquid media where the UCU plays the catalytic role; entrapping the UCU inside a lipid vesicle membrane, which is essentially a selectively permeable membrane; and entrapping CAT and UCU together inside the membrane to provide more substrates of UCU (i.e., oxygen). As observed in Fig. 1, the ESU system serves as a circulating bioreactor, which mainly consists of a high molecular UCU, a lipid enzymosome membrane and an internal liquid microenvironment where the catalyzed reactions take place^20^. The lipid enzymosomal membrane is usually regarded as a differentially permeable membrane similar to a biomembrane, so it is able to locate the UCU of high molecular weight (140 kD) inside the bioreactor and separate the UCU from external environmental bloodstream^17^,^18^. Conversely, the low molecular substrates of UCU (i.e., uric acid) can enter the membrane and the resulting catalysates (such as allantoin and hydrogen peroxide) can leave the bioreactor. Of note, one catalysate of UCU (hydrogen peroxide) is the sole substrate of CAT (also a therapeutic enzyme)^21^, whereas the resulting catalysate of CAT (oxygen) is in turn one substrate of UCU. If both UCU and CAT are simultaneously entrapped inside one enzymosome, this should be a novel favorable delivery system (i.e., ESUC) than ESU alone. Moreover, the alkaline buffer is critical and important as it is the only constituent of the internal microenvironment of a UCU delivery system and the effect of environmental pH on the enzyme activity is extremely significant. As we know, the activities of an enzyme may be influenced by the buffer type because the configurations of an enzyme in different buffers are changeable. For a UCU, borate buffer is most widely used; however, this does not mean it is the best option. Obviously, further efforts need to be made before we can judge which buffer is ideal for an ESU or ESUC. In this study, the critical role of the buffer in enhancing uricolytic activity of UCU in single or complex alkaline enzymosomes was systematically investigated. As presented in Table 1, four buffers, i.e., tris-hydrochloric acid buffer (buffer-A), boric acid-borax buffer (buffer-B), tricine-sodium hydroxide buffer (buffer-C) and bicine-sodium hydroxide buffer (buffer-D) were chosen to make up the internal microenvironments of the UCU delivery systems. ESU or ESUC made of these four different buffers were called ESU-A, ESU-B, ESU-C, ESU-D, ESUC-B and ESUC-D, respectively. Buffer-A is commonly used in biochemical and medical enzyme systems^22^; buffer-B is most widely used for UCU or its enzymosome ESU^18^; buffer-C and buffer-D are “Good buffers” which are reported able to retain well inside biomembranes and particularly suitable for biological systems^23^. The Buffer-C and buffer-D are hydrogen buffers, which are N-substituted amino acids compatible with common biological media^24^.

We demonstrate for the first time that microenvironmental modulation has positive effects on enzyme therapy. The favorable microenvironment is conducive to establishing the best single/complex alkaline enzymosomes with markedly improved stabilities and therapeutic efficacies of an enzyme UCU. Previously, microenvironment modulation was found to have effects on other therapies except enzyme therapy, such as stem cell therapy for spinal cord injury pain^25^, anticancer chemotherapy for enhanced efficacy^26^, and oncolytic virus therapy for increased therapeutic effects^27^. Moreover, microenvironment modulation was also found able to improve the enzymatic catalysis in cephalosporins synthesis for penicillin G acylase^28^.

Our study is the first time to investigate whether favorable microenvironmental changes induce better biochemical and biological features of the single/complex enzymosomes. It is also the first time that the synergetic effects of the buffer type, lipid membrane and UCU-associated enzyme on the efficacies of a therapeutic enzyme UCU have been reported. Moreover, our results first indicate that two correlative therapeutic enzymes can be simultaneously entrapped into one enzymosome to achieve added treatment efficacy. The UCU single/complex enzymosomes in the right buffer significantly can enhance the enzymatic activity, and this has great clinical significance in reducing UCU dosage and adverse enzyme events while increasing the clinical efficacy. Furthermore, research into microenvironmental changes of UCU in single/complex enzymosomes contributes to our new understanding of an effective enzyme therapy strategy to cure diseases. The development of single/complex alkaline UCU enzymosomes in suitable buffers provides valuable tactics in hypouricemic therapy and enzymosomal application.

## Results

### Preparation and characteristics of ESU and ESUC

**Figure 1:**
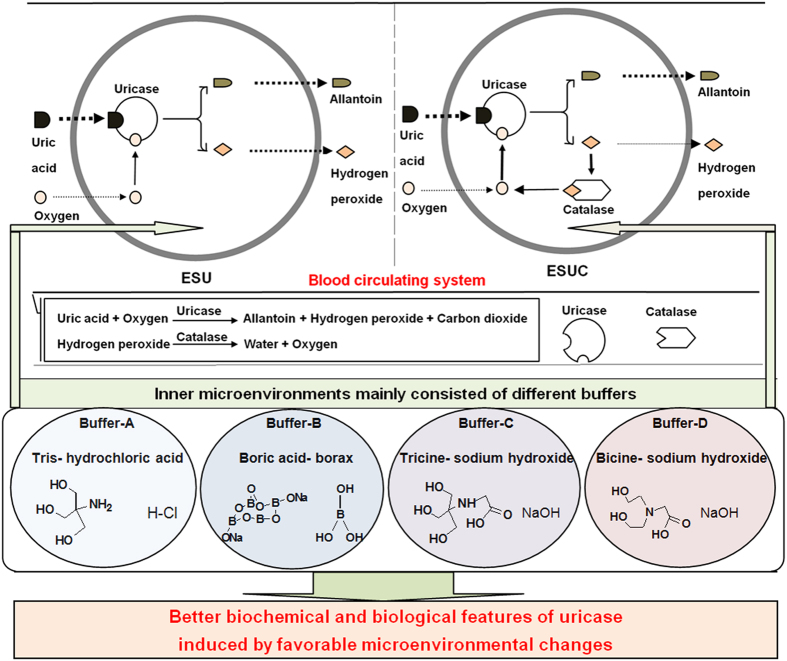
Schematic diagrams of how ESU/ESUC in different buffers changed the biochemical and biological features of UCU as the microenvironments changed.

**Figure 2:**
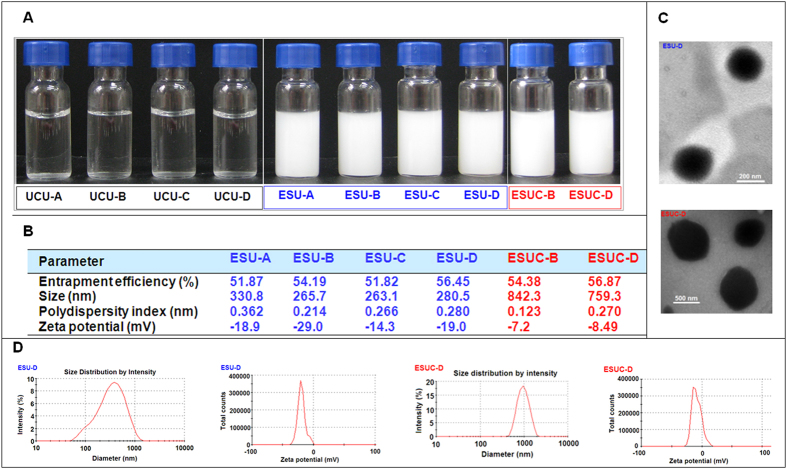
Principal characteristics of ESU and ESUC. (**A**) Optical photographs of UCU, ESU and ESUC; (**B**) Principal parameters of ESU and ESUC (mean, n = 3); (**C**) Transmission electron photomicrographs of ESU-D (bar: 200 nm) and ESUC-D (bar: 500 nm); (**D**) Graphs depicting size distribution and zeta potential of ESU-D and ESUC-D. The maximum activity of free UCU-A was taken as 100%.

The schematic diagrams of ESU/ESUC in different buffers were shown in Fig. 1. The ESU and ESUC were successfully prepared with the entrapment efficiencies ranging from ~52%–~57%; the size of ESU ranged from 260 nm–330 nm, whereas the size of ESUC showed a sudden increase to 750–850 nm (Fig. 2A). Both ESU and ESUC were negatively charged (−7 mV–−29 mV) (Fig. 2B,D). Spherical or sphere-like ESU and ESUC were separately dispersed in the buffer solutions (Fig. 2C).

### Optimum temperature and pH of ESU and ESUC

**Figure 3:**
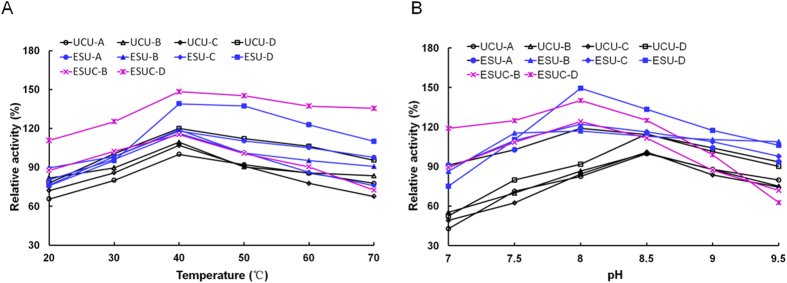
The optimal temperature and pH of UCU, ESU and ESUC. The effects of temperature (**A**) and pH (**B**) on the activity of UCU, ESU and ESUC. The maximum activity of free UCU-A was taken as 100%. The data were presented as mean value, n = 3. Standard deviation (SD) was lower than 5% (data not shown).

All UCU, ESU and ESUC had the same optimum temperature of 40 °C (Fig. 3A). An enzyme’s optimum temperature is the temperature at which the enzyme-catalyzed reaction is the fastest. UCU in buffer-D or simultaneously in enzymosomes had the higher catalytic activities than those in other buffer, it was deduced that the formers had the more favorable configurations, because UCU was a protein in essence. In addition, UCU had an optimum pH of 8.5, whereas ESU and ESUC had the same optimum pH of 8.0 (Fig. 3B). An enzyme’s optimum pH is the acidity-alkalinity degree at which the enzyme-catalyzed reaction is fastest. However, at the same pH value, the enzyme in the suitable type of buffer usually has higher activity than in other types of buffer; for example, Staphylococcus sp. WL1 lipase had the highest activity at optimal pH 7.0 in phosphate buffer but not in tris buffer^29^. In our study, free UCU-D had the highest activity at optimal pH 8.0 in buffer-D but not in buffer-A to -C. Buffer-D was found to be the best buffer for UCU over buffer-A to -C. The different catalytic activity might be caused by the difference in the buffer ion type and concentration, which had influences on enzyme configuration, substrate dissociation and enzyme-substrate binding.

### Stability of ESU and ESUC

**Figure 4:**
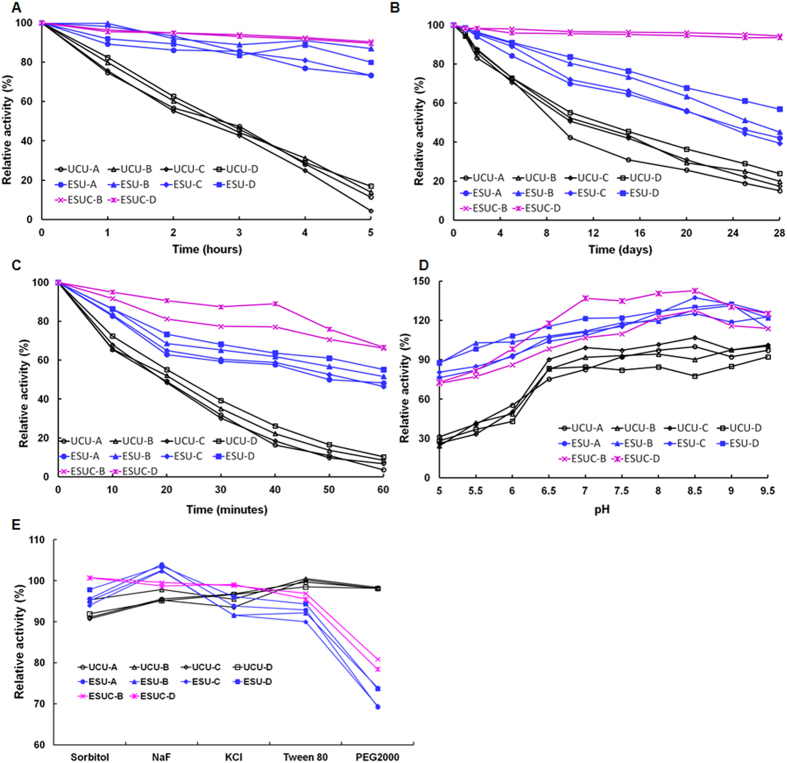
The stabilities of UCU, ESU and ESUC. The effects of (**A**) high temperature (55 °C, thermal stabilities), (**B**) low temperature (4 °C, hypothermal stabilities), (**C**) trypsin (proteolytic stabilities), (**D**) acidity-alkalinity (pH stabilities) and (**E**) chemical agents (chemical stabilities) on the UCU activities. In Figure A–C and E, the original activity of UCU, ESU or ESUC was taken as 100%; in Figure D, the remaining activity of UCU-A at pH 8.5 was taken as 100%. The data were presented as mean value, n = 3. Standard deviation (SD) was lower than 5% (data not shown).

Figure 4A–D showed that ESUC had better thermal (55 °C), hypothermal (4 °C), proteolytic (treated with trypsin), acid-base (pH range from 6–9.5) and chemical (sorbitol, NaF, KCl, Tween 80 and PEG 2000) stabilities than free UCU and ESU. In addition, UCU-D had higher thermal, hypothermal and proteolytic stabilities than did UCU-A to -C; ESU-D had higher thermal, hypothermal and proteolytic stabilities than did ESU-A to -C; and ESUC-D had significantly increased thermal, hypothermal, proteolytic and acid-base stabilities than did UCU, ESU and ESUC-B. All of these might be related to the different existence states of UCU in different buffers. The enzymosomal membranes of ESU or ESUC separated UCU from external environment, which enhanced their stabilities over free UCU. Furthermore, ESUC had higher activities and stabilities than ESU, partly because ESUC acquired more substrates of UCU (oxygen, the catalysate of CAT) and depleted peroxide (the catalysate of UCU and the substrate of CAT) faster, since UCU and CAT co-existed in the same enzymosomal membranes of ESUC. Huang *et al.* reported that the pH value had obvious effects on the stability of free UCU^30^. The chemical nature of UCU was protein, and so its conformations and properties were easier to be influenced by the external situations such as temperature, pH, ions and chemical substances. Among the five chemical agents, sorbitol had the maximal effects on free UCU; PEG 2000 had the maximal effects on ESUC and ESU. Oksanen *et al.* previously reported that the initial substrate protonation state of UCU was the main reason for inhibition by a chloride anion^31^. Here, the inhibition of KCl could be decreased by entrapping UCU together with CAT in enzymosomes. Similarly, sorbitol and NaF could inhibit the catalytic reactions of UCU, whereas inhibition could be retarded by incorporating UCU into the enzymosomes. The inhibition of UCU might be related to the competitive reaction of chemical substances with uric acid at the dioxygen site and the substrate-binding site of UCU, as reported by Gabison *et al.*^32^. On the other hand, it was surprising to find that Tween 80 and PEG 2000 had much stronger inhibitory effects on ESU and ESUC than on free UCU. It was previously reported that the conjugation of UCU with linear PEG was accompanied by loss of enzymatic activity^33^.

### Interaction of UCU with enzymosome membranes

**Figure 5:**
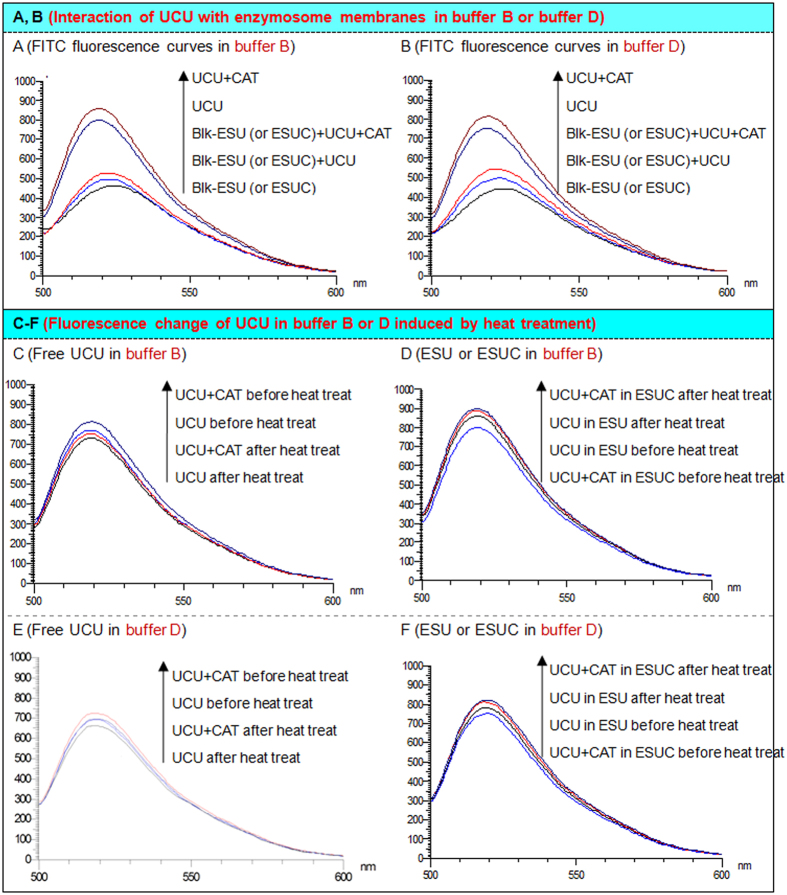
FITC fluorescence changes of UCU. (**A**) Interaction of UCU (in the absence or presence of CAT) with enzymosomal (ESU or ESUC) membranes in buffer-B; (**B**) Interaction of UCU with enzymosome membranes in buffer-D. (**C**) Fluorescence change of UCU (in the absence or presence of CAT) in buffer-B induced by heat treatment; (**D**) Fluorescence change of ESU (or ESUC) in buffer-B induced by heat treatment. (**E**) Fluorescence change of UCU (in the absence or presence of CAT) in buffer-D induced by heat treatment; (**F**) Fluorescence change of ESU (or ESUC) in buffer-D induced by heat treatment. The UCU or CAT concentration was fixed at 100 μg/mL. The FITC concentration was fixed at 0.51 μg/mL. UCU + CAT: mixture of UCU and catalase; Blk-ESU (or ESUC): blank ESU or blank ESUC, the blank ESU was exactly the same as blank ESUC, they were both blank enzymosomes; Blk-ESU (or ESUC) + UCU: mixture of blank enzymosome with free UCU; Blk-ESU (or ESUC) + UCU + CAT: mixture of blank enzymosome with free UCU and CAT;

We detected the interactions of UCU with enzymosome membranes by a FITC fluorescence intensity method. The fluorescence changes indicated the changed conjugate situations of the enzymosomes with FITC or UCU. Our results showed that buffer type had an effect on the interaction extent of UCU or UCU in the presence of CAT with the enzymosomes: UCU or UCU in the presence of CAT interacted with the enzymosomal membranes in buffer-D, albeit to a higher degree than that in buffer-B (Fig. 5A,B). It was previously reported that the FITC, UCU and CAT molecules competitively interacted with the interfacial regions of zwitterionic enzymosomal membranes through electrostatic and hydrophobic interactions^9^,^34^. Corvo *et al.*^35^ recently reported that superoxide dismutase (SOD) could covalently attach to the outer surfaces of enzymosomes, and this type of enzymosomes showed earlier therapeutic activities than did both free SOD and another type of enzymosomes with SOD encapsulated in the aqueous interior. The UCU molecules existed in the ESU or ESUC in three ways: (1) The UCU or UCU in the presence of CAT was attached the outer surface of ESU or ESUC, which in clinical practice might, enable rapid enzyme efficacy for patients; (2) The UCU or UCU in the presence of CAT was attached the inner surface of ESU or ESUC; and (3) the UCU or UCU in the presence of CAT was just entrapped inside the ESU or ESUC without attaching to the inner surfaces of membranes. The sustained enzyme activities confirmed by *in vivo* kinetic tests might mainly ascribe to the last two types of UCU-existing states. The enzyme liposomal membranes might have two functions. One was that the membrane restricted UCU-D mainly inside the internal liquid environment consisting of buffer-D with optimal pH value. It was shown that ESU-D had higher activity than did ESU-A to -C and ESUC-D had higher activity than did ESUC-B. Another function was that the membrane separated large molecular UCU from external environment, which enhanced ESU-D or ESUC-D thermal, hypothermal, proteolytic and acid-base stabilities over free UCU-D.

### Estimation on conformational changes induced by heat treatment

To determine whether heat treatment induced different degrees of conformational change of UCU in different buffers, the fluorescence spectra of UCU-B and UCU-D with the fluorescent probe FITC were detected separately. FITC was conjugated with UCU through the primary amine residue of the amino acid such as lysine. The fluorescence intensity of the conjugation of UCU-FITC changed accordingly when the microenvironments of the aromatic groups changed, followed by changed conformations of UCU. The UCU-FITC in hydrophobic microenvironments had higher fluorescence intensities than that in the hydrophilic microenvironments. After heat treatment, the fluorescence intensities of UCU in the presence of CAT decreased almost the same as that of UCU alone, whereas the fluorescence intensities of UCU released from ESUC increased much more than that of UCU from ESU (Fig. 5C–F). Similar phenomena had been observed in buffer-B and -D, albeit to different degrees. The fluorescence intensity changes suggested that: UCU-D as well as of UCU-B had the same intermolecular interaction change occurred; UCU-D showed less conformational changes in the presence of CAT than UCU-B in the presence of CAT; UCU-D in ESU-D showed less conformational changes than did free UCU-D; UCU-D in ESUC-D showed less conformational changes than did UCU-B in ESUC-B. It was suggested that the microenvironments and conformations of UCU were mainly influenced by the buffer type, enzymosome entrapment, and the co-existence of UCU and CAT. These changes were the comprehensive results of three major interactions: the inter-subunit interaction of UCU or CAT, the intermolecular or intertetramer interaction of UCU or CAT, and the interaction of the UCU or CAT with the enzymosomal membranes^10^. The microenvironments of the aromatic groups of UCU became more hydrophobic due to intermolecular interactions^9^ after heat treatment. Caves *et al.* previously reported that the thermal inactivation of UCU was related to the conformational change, i.e., the loss of helical structure and tertiary structure^36^.

### Estimation on conformational change induced by different buffer and/or enzymosome

The microenvironmental and conformational changes of ESU/ESUC were investigated via fluorescence, circular dichroism spectroscopy, native gel electrophoresis and electrical conductivity.

**Figure 6:**
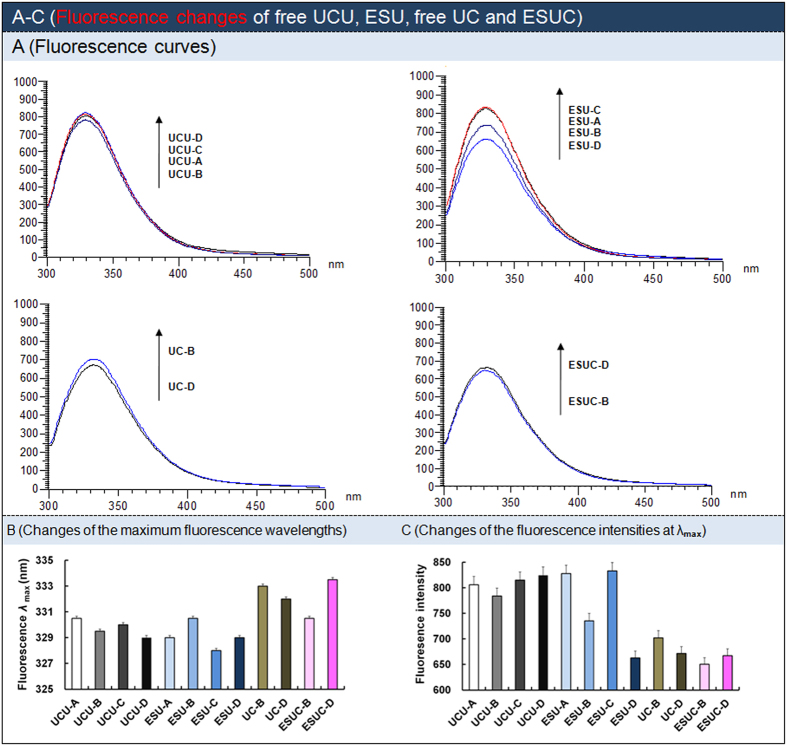
Fluorescence changes of free UCU, ESU, free UC and ESUC. (**A**) Fluorescence, (**B**) Changes of the maximum fluorescence wavelengths. (**C**) Changes of the fluorescence intensities determined at the maximum wavelengths. Free UC: mixture of free UCU and CAT. The data were shown as mean ± SD. n = 3.

The UCU containing some aromatic amino acids in one microenvironment (usually existing in the inner hydrophobic core of a native enzyme) had higher fluorescence intensities and lower maximum wavelengths than did UCU in other microenvironments (such as exposed to outer hydrophilic surfaces). The gradual decrease of the fluorescence intensities (Fig. 6A–C) of UCU at their separate maximum wavelengths suggested that the movements of the primary amine residue of amino acid in UCU from inner hydrophobic cores to outer hydrophilic surfaces in the order of UCU in free UCU, ESU, UCU in the presence of CAT, and ESUC. ESUC-D had the highest wavelength and lowest fluorescence intensity compared with free UCU, ESU and/or ESUC-B, indicating that more primary amine residues of amino acids in ESUC-D existed in the outer hydrophilic surfaces. The flexibility of the hydrophobic part of UCU was reported to be essential for the catalyze reaction and related to the catalytic efficiency^37^.

**Figure 7:**
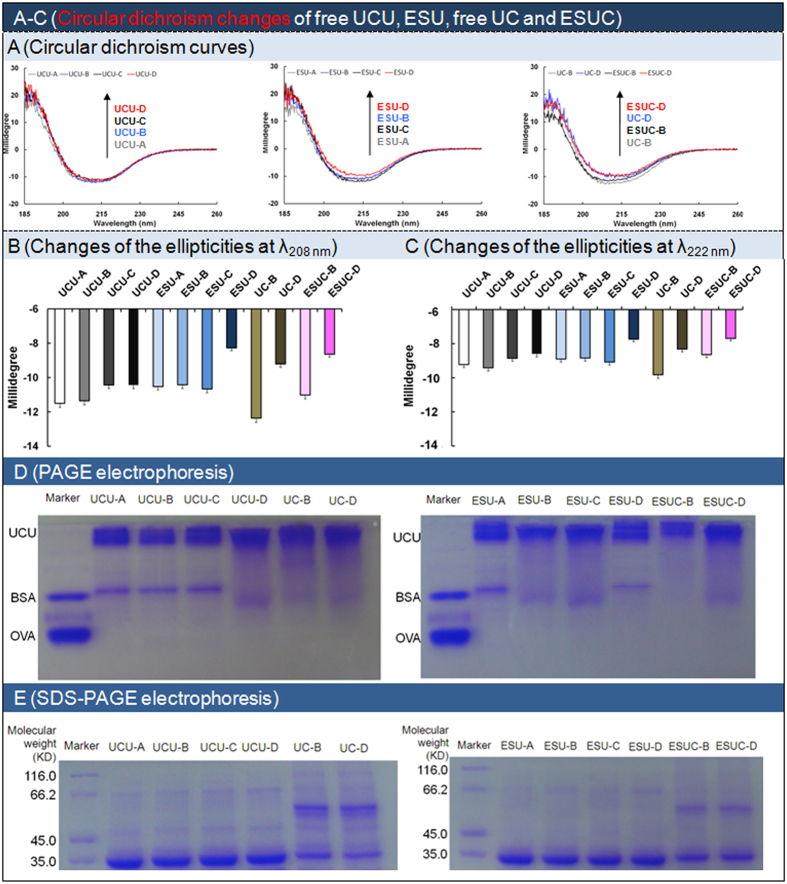
Circular dichroism and gel electrophoresis of free UCU, ESU, UC and ESUC. (**A**) Circular dichroism curves. (**B,C**) Changes of the circular dichroism millidegree at the wavelength of 208 nm and 222 nm. (**D**) Polyacrylamide gel electrophoresis. (**E**) Sodium dodecyl sulfate-polyacrylamide gel electrophoresis. Free UC: mixture of free UCU and CAT. BSA: bovine serum albumin; OVA: ovalbumin. The molecular weights of BSA and OVA were 66 kD and 45 kD, respectively.

Circular dichroism refers to the differential absorption of left and right circularly polarized light. The circular dichroism spectral changes could be induced by 3-D dimensional structure changes of the optically active chiral UCU enzyme molecules. The improvements of the circular dichroism of ESU-D and ESUC-D at 208 nm and 222 nm (Fig. 7A–C) might be explained as follows: ESU-D might have more α-helices than did ESU-A to -C, and UCU-A to -D, and ESUC-D had more than did ESUC-B and other UCU formulations^38^; the differences might be partly due to the different microenvironment (such as polarity and constituent), the different buffer type and/or the influence of the other enzyme CAT and/or the system errors of the analytical instrument (a MOS-450 CD spectrophotometer, Bio-Logic) and analytical method. Buffer type, enzymosome membrane, and the co-existence of UCU and CAT showed the effects on the 3-D dimensional structures of UCU. Furthermore, since it was the first time to investigate the synergetic effects of the buffer type, lipid membrane and UCU-associated enzyme on the circular dichroism changes of the therapeutic enzyme, more efforts should be make before we could better explain the above observed phenomena (the shift of circular dichroism values at 208 nm and 220 nm).

Polyacrylamide gel electrophoresis (PAGE) was used to investigate the changes of noncovalently bonded dimers of UCU. Both UCU and CAT are tetramers, and their dimers are 70 kD and 125 KD, respectively. The native PAGE (Fig. 7D) showed that UCU and its enzymosomes in buffer-D existed mainly in tetramer form. On the other hand, although UCU-B and -C had the dimer bands at 70 kD, these bands disappeared when the UCU-B and -C were separately entrapped into their enzymosomes, which indicated that UCU-B and -C in enzymosomes remained mainly in tetramer forms. Buffer-D, lipid vesicle loading and the addition of CAT were favorable to keep the intact tetramer form of UCU.

Electrical conductivity refers to the reciprocal of the resistivity, which is defined as the current-induced electric field at a point in the conductor or semi-conductor divided by the current density. As shown in Table S2 (Supporting Information), there were obvious differences among the conductivities of free UCU, UC, ESU and ESUC in buffers-A to -D, reflecting the different microenvironmental electricity situations of UCU or its enzymosomes in different types of buffer. UCU-D produced lower electrical conductivity than UCU-A and -B, indicating the lower electricity situations of the microenvironments. Similarly, ESU-D or ESUC-D produced lower electrical conductivity than UCU-D or UC-D, indicating the lower electricity situations of the microenvironments of UCU-D in the single/complex enzymosomes. Buffer-D, lipid vesicle loading and the addition of CAT were favorable to remain the low electricity situations of UCU.

### Unchanged molecular and chemical structure

We investigated the molecular and chemical structure changes of UCU using denaturant gel electrophoresis^39^, sodium dodecyl sulfate (SDS)-PAGE, according to methods described previously. It was obvious that the UCU (in the absence or presence of CAT) or its ESU (or ESUC) had the same bands at 35 kD (the subunit molecular weight of UCU) and/or 62.5 KD (the subunit molecular weight of CAT) (Fig. 7E), which indicated that there were no changes in the molecular and chemical structures of UCU in different types of buffer and enzymosomes. Buffer type and lipid membrane had no effect on the subunit of UCU.

### *In vivo* catalyzed activity of UCU/CAT in enzymosomes

**Figure 8:**
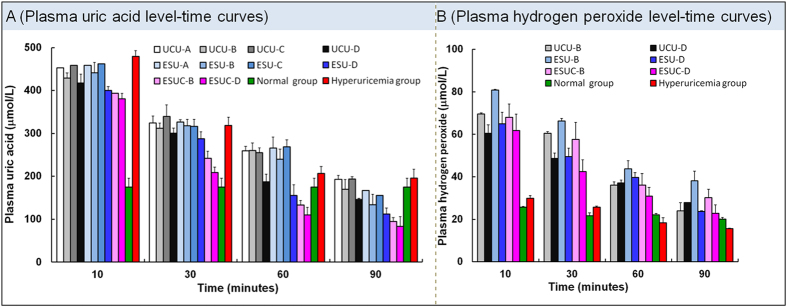
Pharmadynamics of free UCU, ESU and ESUC. (**A**) Plasma uric acid or (**B**) hydrogen peroxide concentration versus time profiles after intravenous injection of UCU, ESU and ESUC. The data were shown as mean ± SD. n = 6 mice per group.

The therapeutic effects of UCU in hyperuricemia mice were demonstrated by its uric acid-lowering capacity^40^,^41^. It took the least time for ESUC-D to lower the plasma uric acid concentration from the same high level to the normal level of mice compared with other UCU formulations (Supporting Figure S1). ESUC-D had the best therapeutic effect of the UCU formulations (Fig. 8A), which was the synthetic result of several factors: (1) the right buffer for UCU was chosen. UCU, ESU and ESUC in buffer-D were better at lowering-uric acid than these formulations in other buffers; (2) the lipid enzymosome entrapment increased the cure efficacy; for example, ESU-D had better lowering-uric acid effect than UCU-D; (3) the simultaneous entrapment of CAT with UCU was beneficial and created higher cure efficacy: when UCU was used together with CAT (Fig. 8B), plasma hydrogen peroxide increased to a lesser degree than UCU was used alone, meanwhile, the plasma uric acid decreased much more. CAT catalyzed hydrogen peroxide (the product of UCU) to produce oxygen (the substrate of UCU), which made the catalytic reaction of UCU go faster. In essence, we could recognize the changed microenvironment of UCU where played the catalyze role to be an important determinant of the enhanced therapeutic response of ESUC-D. In clinics, an UCU formulation was usually intravenously administered. However, a UCU biohybrid hydrogel was subcutaneously implanted into mice to counteract flares of uric acid *in vivo* in 2013^41^. In addition to enzyme therapy and chemotherapy, herbal therapy (using herbal extracts such as Davallia formosana extracts^42^ and Rhizoma Dioscoreae septemlobae extracts^43^) had recently been evaluated in hypertensive mice. Uricosuric drugs increase the renal clearance of uric acid and therefore control hyperuricemia^44^. Further studies might combine several therapy methods to achieve synthetic effects.

### *In vitro* and *in vivo* enzymatic kinetic estimation

**Figure 9:**
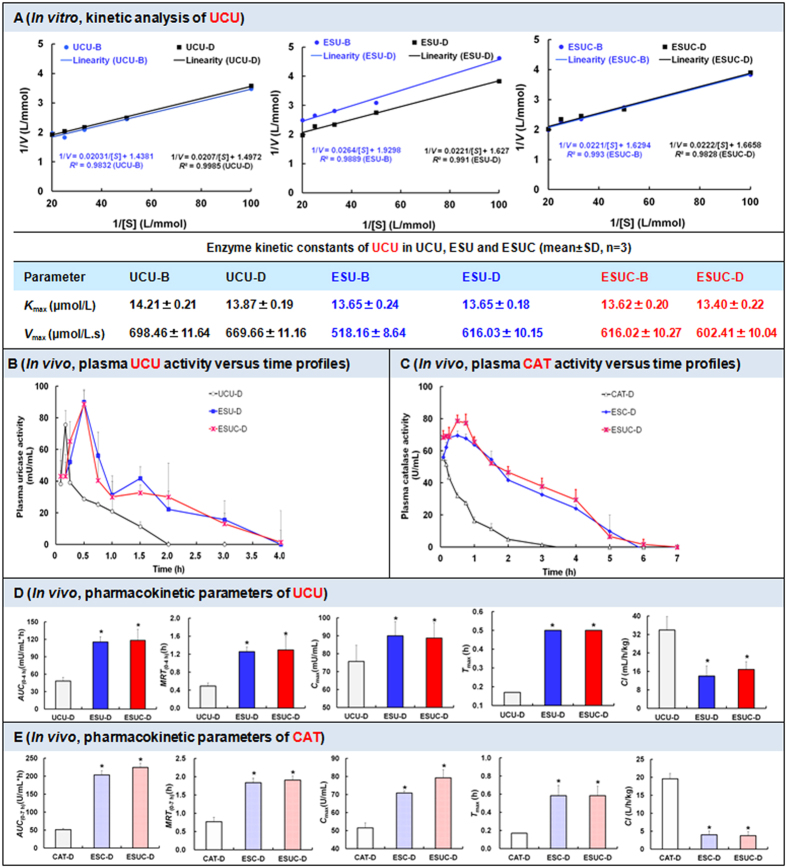
The *in vitro* and *in vivo* kinetic characteristics of UCU, ESU and ESUC. (**A**) Enzymatic kinetic characteristics of UCU, ESU and ESUC: Lineweaver-Burk profiles and Enzyme kinetic constants of UCU, ESU and ESUC (n = 3); (**B**) Plasma UCU activity versus time profiles after intravenous injection of UCU-D, ESU-D and ESUC-D at the same dose (2000 mU/kg of UCU). (**C**) Plasma CAT activity versus time profiles after intravenous injection of CAT-D, ESC-D and ESUC-D at the same dose (1000 U/kg of CAT). (**D**) Main pharmacokinetic parameters of UCU-D, ESU-D and ESUC-D. (**E**) Main pharmacokinetic parameters of CAT-D, ESC-D and ESUC-D. The data were shown as mean ± SD. n = 6 rats per group. *P<0.05 indicated significant differences between ESUC-D (or ESU-D, ESC-D), free UCU-D or free CAT-D.

We determined the *in vitro* kinetic constants of UCU using classic enzyme kinetic analysis^18^. ESUC-D had more favorable enzymatic kinetic properties than did other UCU formulations. *In vitro*, a slightly lower *K*_m_ of ESUC-D (Fig. 9A) suggested that UCU inside the ESUC-D had a slightly higher affinity for the substrate than the other UCU formulations. As previously estimated by Tan *et al.*^18^, the *K*_m_ demonstrated the synthetic functions of the favorable confirmation change of UCU, the charge effects of UCU and its enzymosomes, the interaction of UCU and the enzymosomal membranes, the substrate concentration of UCU (i.e., uric acid level), etc. *In vivo*, enzymatic activity of UCU-D increased greatly by being entrapped into enzymosomes (Fig. 9B). The high activities of ESU-D and ESUC-D remained in rats much longer than did UCU-D, which was demonstrated by the ~2.6-fold *MRT* values (Fig. 9D). The long-term UCU activities of ESU and ESUC might partly ascribe to the UCU-existing states (UCU might conjugate to the internal and external interfaces of the enzymosomal membranes). The relative bioavailabilities of ESU-D and ESUC-D were ~240% and ~245% by separately comparing their *AUC* values with that of free UCU-D. The higher uricolytic activities and the longer circulating times of ESU-D and ESUC-D assured that they had better anti-hyperuricemia effects than did UCU-D. Similarly, the CAT activities in rats treated with CAT in enzymosomes remained at higher levels than those in rats treated with free CAT at every corresponding time point (Fig. 9C). The *MRT* values of ESC and ESUC were 2.39 times and 2.48 times that of free CAT, and the relative bioavailabilities of ESC and ESUC to CAT increased to ~399% and ~440%, respectively (Fig. 9E). The ESU/ESUC and UCU (or ESC/ESUC and CAT) are not bioequivalent (Supporting Table S2).

### Hemolysis test

Free UCU-B and ESU-D had lower hemolysis than that of ESUC-B, UCU-D, ESU-B, and ESUC-D (Supporting Figure S2). By eye, free UCU, ESU and ESUC did not cause hematolysis and erythrocyte accumulation at 37 °C. It was reported that paclitaxel liposomes induced ~11% hemolysis, and that the paclitaxel injection on the market induced ~38% hemolysis^45^. Therefore, ESUC was safe to be injected.

## Discussion

The development of better nanosized therapeutic enzymosomes represents a valuable tactic in an effective enzyme therapy that is, totally different from traditional chemotherapy and newly emerging gene therapy. Research into microenvironmental and conformational changes of enzymosomes has contributed to a new understanding of an effective enzyme therapy strategy to cure diseases. In this study, we have developed UCU/(UCU and CAT) alkaline enzymosomes (ESU/ESUC) for efficient UCU delivery to cure hyperuricemia. Compared to free UCU and ESU, the ESUC in bicine buffer (ESUC-D) had the best biochemical and biological features, including improved thermal, hypothermal, acid-base and proteolytic stabilities, *in vitro* and *in vivo* kinetic characteristics, and uric acid lowering effects. The improved characteristics of ESUC-D were induced by favorable microenvironmental and conformational changes of UCU using three methods: choosing a bicine buffer for UCU, entrapping UCU into a lipid membrane, and entrapping CAT together with UCU. The favorable microenvironmental and conformational changes of ESUC-D were investigated by fluorescence spectroscopy, circular dichroism, native gel electrophoresis and electrical conductivity. The molecular and chemical structure changes of UCU were investigated by denaturant gel electrophoresis. ESUC-D may be a useful nanocarrier of UCU, and complex alkaline enzymosomes may be a good choice for a therapeutic enzyme. There are still a lot of works to do before we can successful make use of ESUC in clinics. Further studies are necessary to evaluate the long-term storage stability and the conformational changes of ESUC, the immunogenicity and enzyme resistance upon repeated administration of ESUC, etc.

## Methods

### Preparation of ESU and ESUC

UCU from the *Candida utilis* species (activity of 4.9 units/mg powder at 25 °C), CAT from bovine liver (activity of 2000–5000 U/mg powder at 25 °C), uric acid and fluorescein isothiocyanate (FITC) were obtained from Sigma-Aldrich (St. Louis, MO, USA). Phospholipid (soybean lecithin, Lipoid S 100) was obtained from Lipoid GmbH (Ludwigshafen, Germany). Cholesterol was obtained from Tianma Fine Chemical Plant (Guangzhou, China). Tris was obtained from Aoboxing Biotech Co., LTD (Beijing, China). Hydrochloric acid, boric acid and sodium hydroxide were obtained from Chuandong Chemical (Group) Co., LTD (Chongqing, China). Borax was obtained from Qiangshun Chemical Co., LTD (Shanghai, China). Tricine and bicine was obtained from Amresco Inc (Solon, OH, USA). ESU was prepared using a modified evaporation method^18^. Firstly, phospholipid and cholesterol (at a molar ratio of 1:1) were dissolved in 30 mL methylene chloride followed by being evaporated away. The formed uniform film was redissolved in 30 mL diethyl ether and added with 10 mL of 50 mmol/L buffer of pH 8.5 (containing 14.29 nmol UCU; buffer-A to buffer-D were used to prepare ESU-A to ESU-D, respectively). The mixture was subjected to sonication and then to evaporation with a hypobaric drying method. The resulting suspension fluid was diluted with equal amounts of corresponding buffer solutions (buffer A to buffer D were added to prepare ESU-A to -D, respectively). ESUC was prepared in a similar way to prepare ESU, the difference was that buffer B (or buffer D) containing enzyme mixtures (14.29 nmol UCU and 8 nmol CAT) instead of only UCU was used, respectively. ESC was prepared in a similar way to prepare ESU, but the difference was that only CAT instead of UCU was used.

### Principal characteristics of ESU and ESUC

The average particle size and zeta potential of ESU (or ESUC) were determined by dynamic light scattering (Zeta-Sizer Nano ZS90 instrument, Malven Instruments Ltd., UK). The sample was prepared by diluting the above produced preparation with 9 times volume of corresponding buffer. The morphology of ESU-D and ESUC-D were observed by the transmission electron microscopy (TEM H-7500, Hitachi, Japan). The samples were prepared by diluting the above produced preparation with 19 times volume of buffer D. The entrapment efficiency of ESU (or ESUC) was determined by dividing the amount of the entrapped enzyme by the total amount of enzyme in the vesicle systems^10^. The entrapped enzyme was separated from its vesicle using a gel exclusion chromatography method (with a 15 mm × 400 mm Sephadex G-200 column). Enzyme content was determined by the Coomassie blue method^18^. The effects of temperature and pH on UCU, ESU and ESUC were investigated in the range of 20 °C–70 °C and pH 7.0–9.5, respectively.

### Optimum temperature and pH of ESU and ESUC

The effects of temperature and pH on ESU (or ESUC) were investigated in the temperature range of 20 °C–70 °C and pH range of 7.0–9.5, respectively. The UCU activity was determined according to a previous study^17^. One U/mL of UCU was defined as the amount of UCU able to oxidize 1 μmol/L of uric acid to allantoin per minute at 25 °C.

### Stability of ESU and ESUC

(1) The effects of temperature on ESU (or ESUC) were determined as follows: ESU (or ESUC, containing 100 μg/mL UCU) was incubated at 55 °C for 1 h or 4 °C for 28 d. The UCU activity was determined at different times^17^. (2) The effects of trypsin on ESU (or ESUC) were determined as follows: ESU (or ESUC) was treated with trypsin at 37 °C, the final concentrations of UCU and trypsin were 100 μg/mL. Aliquots were taken at predetermined times and assayed. (3) The effects of acidity-alkalinity on ESU (or ESUC) were determined as follows: ESU (or ESUC) was incubated with its corresponding buffer with a pH range from 5.0–9.5 at 40 °C for 40 min and then the remaining UCU activity was determined at 25 °C. The final concentration of UCU was 100 μg/mL. (4) The effects of chemical agents on ESU (or ESUC) were determined as follows: ESU (or ESUC) was treated with different chemical agents at 25 °C for 1 h, the final concentrations of UCU, sorbitol, sodium fluoride (NaF), potassium chloride (KCl), polysorbate 80 (Tween 80), polyethylene glycol 2000 (PEG 2000) were 150 μg/mL, 1 mmol/L, 1 mmol/L, 4 mmol/L and 2.5 mmol/L, respectively. The remaining UCU activity was determined in 1 h.

### Measurement of FITC fluorescence change

The interaction of UCU (in the absence or presence of CAT) with ESU (or ESUC) in buffer B (or buffer D) was determined according to the FITC fluorescence method reported previously^19^. Briefly, UCU (100 μg/mL), UCU in the presence of CAT (i.e., the mixture of 100 μg/mL UCU and 100 μg/mL CAT), the blank ESU (or blank ESUC, which is exactly the same as blank ESU) were separately mixed with FITC (25.68 μmol/L) in a volume ratio of 49:1. After separately incubating these mixtures for 5 min in the dark, the excitation wavelength was set at 480 nm and the emission fluorescence intensities were recorded from 500–600 nm using a fluorescence spectrophotometer (F-2500, Shimadzu). The effect of UCU was examined by incubating UCU with blank ESU (or ESUC) for 1 h at 25 °C before adding the FITC solution. Similarly, the effect of UCU in the presence of CAT was examined by incubating the enzyme mixtures with blank ESU (or blank ESUC).

Fluorescence change of UCU (in the absence or presence of CAT) or its ESU (or ESUC) in buffer B (or buffer D) induced by heat treatment was determined according to a modified FITC fluorescence method^10^. Briefly, UCU (100 μg/mL), UCU in the presence of CAT (i.e., the mixture of 100 μg/mL UCU and 100 μg/mL CAT), ESU containing 100 μg/mL UCU and ESUC containing 100 μg/mL UCU and 100 μg/mL CAT were separately incubated at 55 °Cor 25 °C for 3 h. Then, free UCU in the absence or presence of CAT was mixed with FITC and handled in the same way as above, and then the emission fluorescence intensity was recorded from 500–600 nm at the excitation wavelength of 480 nm. For the enzymosome of UCU in the absence or presence of CAT, chloroform was added into the ESU (or ESUC) in a volume ratio of 1:2, and then the mixture was centrifuged at 3000 rpm for 5 min; the supernatant was taken out and tested using the above procedure.

### Fluorescence changes of UCU in different buffer and/or enzymosome

Fluorescence changes of UCU (in the absence or presence of CAT) or its ESU (or ESUC) in buffer-A to -D were determined according to a modified enzyme fluorescence method^46^,^47^. Briefly, UCU (50 μg/mL) or UCU in the presence of CAT (i.e., the mixture of 25 μg/mL UCU and 25 μg/mL CAT) was separately determined. The emission fluorescence intensity was recorded from 300–500 nm at the excitation wavelength of 280 nm. For the enzymosome of UCU in the absence or presence of CAT, chloroform was added and it was tested in the way described above.

### Circular dichroism changes of UCU in different buffer and/or enzymosome

Circular dichroism changes of UCU (in the absence or presence of CAT) or its ESU (or ESUC) in buffer-A to -D was determined using a CD spectrophotometer (MOS-450, Bio-Logic). Briefly, UCU (200 μg/mL) or UCU in the presence of CAT (i.e., the mixture of 100 μg/mL UCU and 100 μg/mL CAT) was separately determined. The emission fluorescence intensity was recorded from 185–260 nm. For the enzymosome of UCU in the absence or presence of CAT, chloroform was added and it was tested in the way described above.

### PAGE and SDS-PAGE of UCU in different buffer and/or enzymosome

PAGE was preformed according to methods described previously^12^. Briefly, equal amounts of free UCU, ESU or ESUC (containing 10 μg of UCU or the mixture of 5 μg of UCU and 5 μg of CAT) were spotted and subjected to electrophoresis (Bio-Rad Laboratories, Hercules, CA, USA). Then, the gel was stained with Coomassie brilliant blue R-250 dye solution for 20 min, and then destained with a destaining solution (methanol:acetic acid:distilled water in a volume ratio of 1:1:8) for 2 d. In a similar manner, SDS-PAGE was performed. The differences were listed as follows: samples were heated in a boiling water bath for 5 min before they were spotted; 10% SDS was added into both the separation gel and spacer gel.

### *In vivo* catalyzed activity of UCU/CAT in enzymosomes

Male Sprague Dawley rats (250 ± 20) g and Kunming strain mice (20 ± 2) g were supplied by the Laboratory Animal Center of Chongqing Medical University (Chongqing, China). All animals were acclimatized at a temperature of 25 ± 3 °C and a relative humidity of 70 ± 5% under natural light/dark conditions for 1 week before dosing. All experimental protocols were approved by the Laboratory Animal Committee, Chongqing Medical University. All animal experiments were performed in accordance with the protocol approved by the Laboratory Animal Committee, Chongqing Medical University.

All male Kunming strain mice (20 ± 2 g) were given uric acid in 5% sodium carboxyl methyl cellulose by intraperitoneal injection at a dose of 250 mg/kg. After 5 min, the mice were given free UCU, ESU and ESUC at the same UCU dose of 2000 mU/kg, respectively. Twelve groups of mice (twenty-four mice per group) were arranged as follows: groups 1–4 were given free UCU-A, UCU-B, UCU-C or UCU-D; groups 5–8 were given ESU-A, ESU-B, ESU-C and ESU-D; groups 9 and 10 were given ESUC-B and ESUC-D, group 11 was a normal mouse group (negative control group); and group 12 was a hyperuricemia mouse model group without treatment (positive control group). The blood samples of six mice in each group were taken from the eye socket at various time intervals (10, 30, 60 and 90 min), and plasma uric acid levels were measured using an assay kit available in the clinical lab (Nanjing Jiancheng Bioengineering Institute, Nanjing, China) after centrifugation (3000 rpm for 10 min) according to previously described method^17^. Hydrogen peroxide levels were detected using the hydrogen peroxide assay kit (Nanjing Jiancheng Bioengineering Institute, Nanjing, China) according to the instruction manual.

### *In vitro* and *in vivo* enzymatic kinetic estimation

The *in vitro* kinetic parameters of UCU were determined by catalyzing the oxidation of uric acid solutions at different concentrations (10, 20, 30, 40 and 50 μmol/L) at 25 °C^18^. The reaction medium contained 100 μg/mL of free UCU or entrapped UCU in its enzymosomes (ESU or ESUC) in buffer B (or buffer D). The enzyme kinetic parameters were calculated by the lineweaver-Burk plotting (Eq. 1).


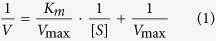


Where *V* was the reaction velocity, [*S*] was the substrate (uric acid) concentration, *K*_m_ was the Michaelis constant, and *V*_max_ was the maximum velocity.

Thirty SD rats (200 ± 20 g) were intravenously administered free UCU-D, ESU-D, ESUC-D, CAT-D, ESC-D at the same UCU dose of 2000 mU/kg and/or CAT dose of 1000 U/kg, respectively. Blood samples were obtained from the posterior orbital venous plexus at predetermined times and subjected to centrifugation for 10 min at 3000 rpm for further analysis. The plasma UCU activity was measured according to a previously described patented kinetic method^17^. The plasma CAT activity was detected using the catalase assay kit (Nanjing Jiancheng Bioengineering Institute, Nanjing, China) according to the instruction manual. The pharmacokinetic parameters and bioequivalence evaluation were calculated using Drug and Statistics software (DAS ver. 2.1.1, Mathematical Pharmacology Professional Committee of China, Shanghai, China).

### Hemolysis test

The rabbit erythrocyte dispersions (2%, W/V) were prepared according to previously reported methods^18^. Then, 2.5 mL of erythrocyte dispersions was separately added to 0.5 mL of test sample (i.e., UCU, ESU or ESUC containing 100 μg/mL UCU and/or 100 μg/mL CAT) and 2.0 mL of saline water. Here, in the case of positive or negative controls, 2.5 mL of distilled water or saline water was added. The mixtures were placed at 37 °C for 1 h, and then subjected to centrifugation at 3000 rpm for 10 min. Then, the supernatant was taken out and determined at 540 nm^46^. The hemolysis rate was calculated by comparing the absorbance of the sample to that of distilled water.

### Statistical analysis

All data are shown as the mean ± standard deviation unless particularly outlined. The Student’s paired t-test was used to calculate significant difference. Statistical significance was established at *P*<0.05. Pharmacokinetic and bioequivalence analyses were conducted using DAS software (Mathematical Pharmacology Professional Committee of China, Shanghai, China).

## Additional Information

**How to cite this article**: Zhou, Y. *et al.* Uricase alkaline enzymosomes with enhanced stabilities and anti-hyperuricemia effects induced by favorable microenvironmental changes. *Sci. Rep.* **6**, 20136; doi: 10.1038/srep20136 (2016).

## Supplementary Material

Supplementary Information

## Figures and Tables

**Table 1:** Molecular formula, weights, structures and dissociation constants of the constitutes in Buffer-A, -B, -C and -D.

Name	Buffer-A		Buffer-B		Buffer-C		Buffer-D	
	Tris- hydrochloric acid buffer		Boric acid- borax buffer		Tricine- sodium hydroxide buffer		Bicine- sodium hydroxide buffer	
	Tris	Hydrochloric acid	Borax	Boric acid	Tricine	Sodium hydroxide	Bicine	Sodium hydroxide
Molecular formula	C_4_H_11_NO_3_	HCl	Na_2_B_4_O_7_	H_3_BO_3_	C_6_H_13_NO_5_	NaOH	C_6_H_13_NO_4_	NaOH
Molecular weight	121.14	36.46	201.22	61.81	179.17	40.01	163.17	40.01
Molecular Structure	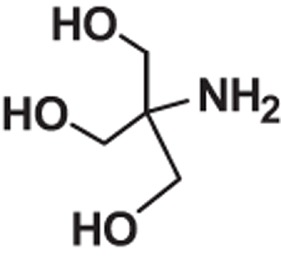	H−CI	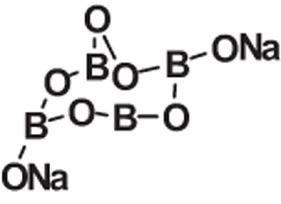	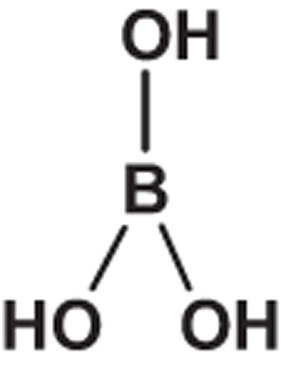	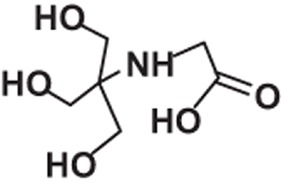	NaOH	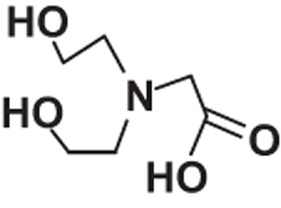	NaOH
p*K*_a_	8.10		9.20		8.15		8.35	

*pK*_a_: dissociation constant.
