# Erratum: Uricase alkaline enzymosomes with enhanced stabilities and anti-hyperuricemia effects induced by favorable microenvironmental changes

**DOI:** 10.1038/srep46390

**Published:** 2017-06-30

**Authors:** Yunli Zhou, Mi Zhang, Dan He, Xueyuan Hu, Huarong Xiong, Jianyong Wu, Biyue Zhu, Jingqing Zhang

Scientific Reports 6: Article number: 20136; 10.1038/srep20136 published online: 01 29 2016; updated: 06 30 2017.

The original HTML version of this Article listed an incorrect volume number. This has now been corrected in the HTML version; the PDF version was correct at the time of publication.

