# Supplementary material for: Uricase alkaline enzymosomes with enhanced stabilities and anti-hyperuricemia effects induced by favorable microenvironmental changes
Source: Sci Rep. 2016 Jan 29;7:20136. doi: 10.1038/srep20136 (PMC4731772; doi:10.1038/srep20136)
Supplement: Supplementary Information [file srep20136-s1.pdf]

Supplementary Information

Uricase alkaline enzymosomes with enhanced stabilities and anti-hyperuricemia effects induced by favorable microenvironmental changes

Yunli Zhou,[1,#] Mi Zhang,[1,#] Dan He[1,#], Xueyuan Hu,[1,#] Huarong Xiong,[1,#] Jianyong Wu,[1] Biyue Zhu,[2] Jingqing Zhang[1,*]

[#] These authors contributed equally to this work.
[*]Correspondence and requests for materials should be addressed to J.Zhang (email: zjqrae01@163.com)

The Supplementary Information includes:

Supplementary Figures S1.

Supplementary Figures S2.

Supplementary Table S1

Supplementary Table S2

*Corresponding author:
Jingqing Zhang, Ph.D., Professor
Medicine Engineering Research Center
Chongqing Medical University
Room 802, Building 9
Shiyoulu Road 1, Yuzhong District
Chongqing 400042, PR China
Phone: (+86) 013320359206
E-mail: zjqrae01@163.com

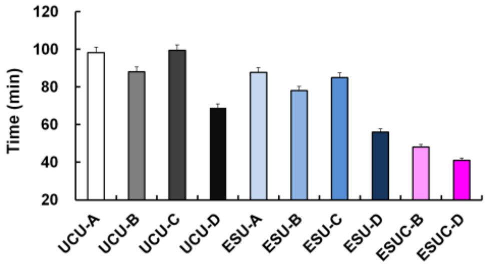

Supplementary Figure S1. Time to achieve the normal uric acid level in mice. Pharmadynamics of free UCU, ESU and ESUC. The data were shown as mean±SD. n=6 mice per group.

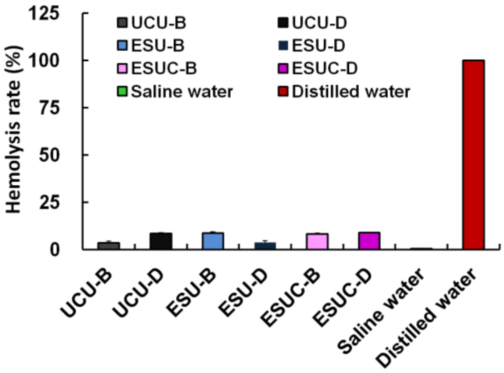

Supplementary Figure S2. Hemolysis rates of free UCU, ESU and ESUC in buffer-B and -D. The data were shown as mean±SD. n=3. The saline water was taken as the negative control. The distilled water was taken as the positive control.

Supplementary Table S1. The electrical donductivities of UCU formulations in different buffer types and ionic strengths.

Formulation	Conductivity (μs/cm)	
	Buffer (50 mM)	Buffer (10 mM)
Buffer-A	2363.33±15.28	597.00±7.55
Buffer-B	1256.33±22.85	264.67±3.51
Buffer-C	1692.33±34.85	382.33±3.06
Buffer-D	1130.00±20.30	324.67±10.07
UCU-A	1919.67±30.02	417.33 ±11.24
UCU-B	1114.33±17.93	162.33 ±8.96
UCU-C	933.33 ±18.50	373.67 ±9.45
UCU-D	1047.33±11.50	276.67±6.51
ESU-A	2113.33±35.12	453.67±5.51
ESU-B	1024.33±8.50	181.67±2.08
ESU-C	1494.33±33.17	338.33±12.86
ESU-D	872.67±13.20	259.67±8.62
UC-B	1240.67±4.51	476.67±5.03
UC-D	1167.00±7.00	492.00±8.19
ESUC-B	986.00±12.53	148.67±3.06
ESUC-D	954.00±23.30	174.67±5.69

Notes: UCU concentration in free UCU or ESU was 100 μg/mL. Both UCU and CA concentration in free UC or ESUC were 50 μg/mL. Data were presented as mean ± standard deviation (n=3).

Supplementary Table S2. Bioequivalence evaluation of UCU and/or CAT formulations after intraveous administration at the same UCU dose of 2000 mU/kg and/or CAT dose of 1000 U/kg.

Formulation 1 and 2	Parameter	90% confidential interval calculated	*P* value calculated	Bioequivalence standard	Bioequivalence
ESUC and UCU	*AUC*	118.7%～131.1%	-	80%-125%	No
	C_{max}	100.4% ～107.7%	-	70%-143%	Yes
	T_{max}	-	＜0.05	＞0.05	No
	In all	-	-	-	No
ESU and UCU	*AUC*	118.8%～129.9%	-	80-125%	No
	C_{max}	99.9%～107.6%	-	70-143%	Yes
	T_{max}	-	＜0.05	＞0.05	No
	In all	-	-	-	No
ESUC and ESU	*AUC*	95.0% ～102.5%	-	80-125%	Yes
	C_{max}	96.9% ～102.5%	-	70-143%	Yes
	T_{max}	-	＞0.05	＞0.05	Yes
	In all	-	-	-	Yes
ESUC and CAT	*AUC*	136.5%～141.7%	-	80%-125%	No
	C_{max}	100.9% ～104.4%	-	70%-143%	Yes
	T_{max}	-	＜0.05	＞0.05	No
	In all	-	-	-	No
ESC and CAT	*AUC*	133.8%～140.1%	-	80-125%	No
	C_{max}	104.7%～107.5%	-	70-143%	Yes
	T_{max}	-	＜0.05	＞0.05	No
	In all	-	-	-	No
ESUC and ESC	*AUC*	96.2% ～100.7%	-	80-125%	Yes
	C_{max}	95.7% ～99.2%	-	70-143%	Yes
	T_{max}	-	＞0.05	＞0.05	Yes
	In all	-	-	-	Yes

Notes: Data presented as mean ± standard deviation (n=6).
